# Tanshinone IIA attenuates neuroinflammation via inhibiting RAGE/NF-κB signaling pathway in vivo and in vitro

**DOI:** 10.1186/s12974-020-01981-4

**Published:** 2020-10-14

**Authors:** Bo Ding, Chengheng Lin, Qian Liu, Yingying He, John Bosco Ruganzu, Hui Jin, Xiaoqian Peng, Shengfeng Ji, Yanbing Ma, Weina Yang

**Affiliations:** 1 Department of Human Anatomy, Histology and Embryology, School of Basic Medical Sciences, Xi’an Jiaotong University Health Science Center, 76 Yanta West Road, Xi’an, 710061 Shaanxi China; 2 Medical Undergraduates of Xi’an Jiaotong University Health Science Center, 76 Yanta West Road, Xi’an, 710061 Shaanxi China

**Keywords:** Alzheimer’s disease, Tanshinone IIA, Amyloid beta, Neuroinflammation, Receptor for advanced glycation end products

## Abstract

**Background:**

Glial activation and neuroinflammation play a crucial role in the pathogenesis and development of Alzheimer’s disease (AD). The receptor for advanced glycation end products (RAGE)-mediated signaling pathway is related to amyloid beta (Aβ)-induced neuroinflammation. This study aimed to investigate the neuroprotective effects of tanshinone IIA (tan IIA), a natural product isolated from traditional Chinese herbal *Salvia miltiorrhiza* Bunge, against Aβ-induced neuroinflammation, cognitive impairment, and neurotoxicity as well as the underlying mechanisms in vivo and in vitro.

**Methods:**

Open-field test, Y-maze test, and Morris water maze test were conducted to assess the cognitive function in APP/PS1 mice. Immunohistochemistry, immunofluorescence, thioflavin S (Th-S) staining, enzyme-linked immunosorbent assay (ELISA), real-time quantitative reverse-transcription polymerase chain reaction (qRT-PCR), and western blotting were performed to explore Aβ deposition, synaptic and neuronal loss, microglial and astrocytic activation, RAGE-dependent signaling, and the production of pro-inflammatory cytokines in APP/PS1 mice and cultured BV2 and U87 cells.

**Results:**

Tan IIA treatment prevented spatial learning and memory deficits in APP/PS1 mice. Additionally, tan IIA attenuated Aβ accumulation, synapse-associated proteins (Syn and PSD-95) and neuronal loss, as well as peri-plaque microgliosis and astrocytosis in the cortex and hippocampus of APP/PS1 mice. Furthermore, tan IIA significantly suppressed RAGE/nuclear factor-κB (NF-κB) signaling pathway and the production of pro-inflammatory cytokines (TNF-α, IL-6, and IL-1β) in APP/PS1 mice and cultured BV2 and U87 cells.

**Conclusions:**

Taken together, the present results indicated that tan IIA improves cognitive decline and neuroinflammation partly via inhibiting RAGE/NF-κB signaling pathway in vivo and in vitro. Thus, tan IIA might be a promising therapeutic drug for halting and preventing AD progression.

## Background

Alzheimer’s disease (AD) is a neurodegenerative disorder that leads to cognitive impairment and behavioral changes [1]. Aberrant amyloid beta (Aβ) accumulation in senile plaques and the hyperphosphorylation of tau protein-forming neurofibrillary tangles (NFTs) are hallmark features of the AD neurodegenerative cascade. Increasing evidence indicates that neuroinflammation exerts vital effects in the pathogenesis of AD [2–4]. Aβ deposition and chronic neuroinflammatory responses interplay with each other to form a vicious cycle that expands the damaging effects [5]. Under inflammatory conditions, both activated microglia and astrocytes can produce and secrete multiple pro-inflammatory cytokines, such as tumor necrosis factor-α (TNF-α), interleukin-6 (IL-6), and interleukin-1β (IL-1β), all of which can confer neurotoxicity [6].

The receptor for advanced glycation end products (RAGE) is a pattern recognition receptor and a member of the immunoglobulin superfamily that recognizes a variety of ligands, including the advanced glycation end products (AGEs) proteins, Aβ, inflammatory mediators, and the adhesion molecule Mac-1 [7, 8]. In the brain, RAGE is expressed on neurons, microglia, astrocytes, and endothelial cells [9–11]. The activation of RAGE by Aβ in the microglia enhances the production of pro-inflammatory cytokines TNF-α, IL-6, and IL-1β, as well as chemokines [7]. In astrocytes and cerebral endothelial cells, the activation of RAGE by Aβ induces oxidative stress by increasing the production of reactive oxygen species [12]. Aβ binding to RAGE on neurons induces the release of macrophage colony-stimulating factor, which then activates microglia [13]. RAGE ligation by Aβ induces a cell signaling cascade that leads to activation of the transcription factor nuclear factor-κB (NF-κB). Activation of NF-κB stimulates the production of various pro-inflammatory cytokines, chemokines, pro-oxidants, and RAGE itself [7, 14]. High levels of RAGE in the brain could exacerbate Aβ-induced neuroinflammation, synaptic and neuronal dysfunction, as well as cognitive impairment [15, 16]. Thus, the reagents aimed at RAGE blockade or suppressing RAGE-mediated NF-κB signaling pathway may have potential therapeutic advantages for AD.

Tanshinone IIA (tan IIA) is an important lipophilic diterpene extracted from *Salvia miltiorrhiza* Bunge. Many experimental and clinical investigations have reported that tan IIA can prevent or slow the progression of a wide spectrum of diseases, including cardiovascular, cancer, cerebrovascular diseases, and AD [17–21]. Our previous studies indicated that tan IIA protected primary neurons from Aβ-induced neurotoxicity [22, 23]. We also confirmed that tan IIA prevented streptozotocin-induced memory deficits in mice [20]. Recently, our experimental data revealed that tan IIA inhibited endoplasmic reticulum stress-induced apoptosis in APPswe/PS1dE9 (referred to as APP/PS1) transgenic mice and SH-SY5Y cells [24, 25], although increasing evidence showed that tan IIA exhibits anti-inflammatory activity both in vivo and in vitro [21, 26–28]. However, the effects of tan IIA on RAGE-mediated neuroinflammation and its underlying molecular mechanisms in AD are not reported. Thus, in the present study, we investigated the efficacy of tan IIA in Aβ accumulation, neuroinflammation, synaptic and neuronal loss, as well as learning and memory capacity in APP/PS1 transgenic mice. Additionally, we further confirmed the anti-inflammatory effects of tan IIA on Aβ-induced neuroinflammation in BV2 and U87 cells respectively. The present results provided evidence that tan IIA could prevent neuroinflammatory responses, at least in part via suppressing RAGE-mediated NF-κB signaling pathway activation in microglia and astrocytes.

## Material and methods

### Drugs and reagents

Dimethylsulfoxide (DMSO), hexafluoro-2-propanol (HFIP), bovine serum albumin (BSA), and thioflavin S (Th-S) were purchased from Sigma-Aldrich (Saint Louis, MO, USA). Aβ_1–42_ was obtained from APeptide Co., Ltd (Shanghai, China). Tan IIA (MR 294.34 of purity > 99%) was purchased from Shanghai YuanYe Biotechnology Corporation (Shanghai, China). Antibodies were from several companies: anti-RAGE and anti-NeuN were obtained from Abcam (Cambridge, MA, USA); anti-ionized calcium-binding adapter molecule 1(Iba-1) was purchased from GeneTex (Alton Parkway Irvine, CA, USA); anti-phospho-IκBα (Ser32), anti-total-IκBα, anti-phospho-NF-κB p65 (Ser536), anti-total-NF-κB p65, anti-synaptophysin (Syn), anti-PSD-95, anti-histone H3, and FITC-conjugated goat anti-rabbit IgG were obtained from Cell Signaling Technologies (Beverly, MA, USA); anti-β-actin was purchased from Santa Cruz (CA, USA); anti-MOAB2, anti-glial fibrillary acidic protein (GFAP), and Cy3-conjugated goat anti-mouse IgG were obtained from Novus Biologicals (Littleton, CO, USA); horseradish peroxidase (HRP)-conjugated goat anti-rabbit and goat anti-mouse IgG were purchased from Pierce Corporation (Rockford, IL, USA) and Zhongshan Golden Bridge Biotechnology Corporation (Beijing, China). Nitrocellulose filter (NC) membrane was purchased from Pall Corporation (New York, NY, USA). Enhanced chemiluminescence (ECL) detection reagents were obtained from Bio-Rad Laboratories, Inc. (Hercules, CA, USA). Mouse Aβ_1–40_ and Aβ_1–42_ enzyme-linked immunosorbent assay (ELISA) kits were purchased from Invitrogen (Carlsbad, CA, USA). Mouse and human TNF-α, IL-6, and IL-1β ELISA kits were obtained from Mei-mian Biotechnology (Yancheng, China). Bay11-7082 was purchased from Enzo (New York, USA). FPS-ZM1 was obtained from Selleck (Houston, Texas, USA). RNAiso Plus, Prime Script™ RT Master Mix, and SYBR® Premix Ex Taq™ II were obtained from Takara Biotechnology Corporation (Dalian, China). Mouse direct polymerase chain reaction (PCR) kit was purchased from Bimake (Houston, Texas, USA). Fetal bovine serum (FBS) was obtained from Biological Industries (Kibbutz Beit Haemek, Israel). Dulbecco’s modified Eagle’s medium (DMEM) was purchased from Shanghai Yuanpei Biotechnology Corporation (Shanghai, China). All other chemicals used were of the highest grade commercially available.

### Animals

APP/PS1 male transgenic mice (B6C3-Tg) and wild-type (WT) female mice with the same genetic background were obtained from the Model Animal Research Center of Nanjing University (N000175, Nanjing, China). Heterozygous APP/PS1 males were bred with WT females. Their offspring were genotyped by PCR using DNA isolated from tail tissues. The specific primers were as follows: APP (forward, 5′-GAC TGA CCA CTC GAC CAG GTT CTG-3′; reverse, 5′-CTT GTA AGT TGG ATT CTC ATA TCC G-3′); PS1 (forward, 5′-AAT AGA GAA CGG CAG GAG CA-3′; reverse, 5′-GCC ATG AGG GCA CTA ATC AT-3′). All experimental mice were group-housed in a controlled environment (22–25 °C, 50% humidity, 12 h light/dark cycle) and received a standard diet and water ad libitum. All experiments were conducted according to the guidelines for animal care and use of China.

### Group design and drug treatment

After genotyping, a total of 30 APP/PS1 transgenic mice (6 months, male) were randomly divided into three groups (*n* = 10 in each group): APP/PS1 group, tan IIA 5 mg/kg group, and tan IIA 20 mg/kg group. Age-matched WT male mice with the same genetic background were used as the control group (*n* = 10). Tan IIA was dissolved in 0.1% DMSO and further diluted to different concentrations in a 0.9% NaCl solution.

**Fig. 1:**
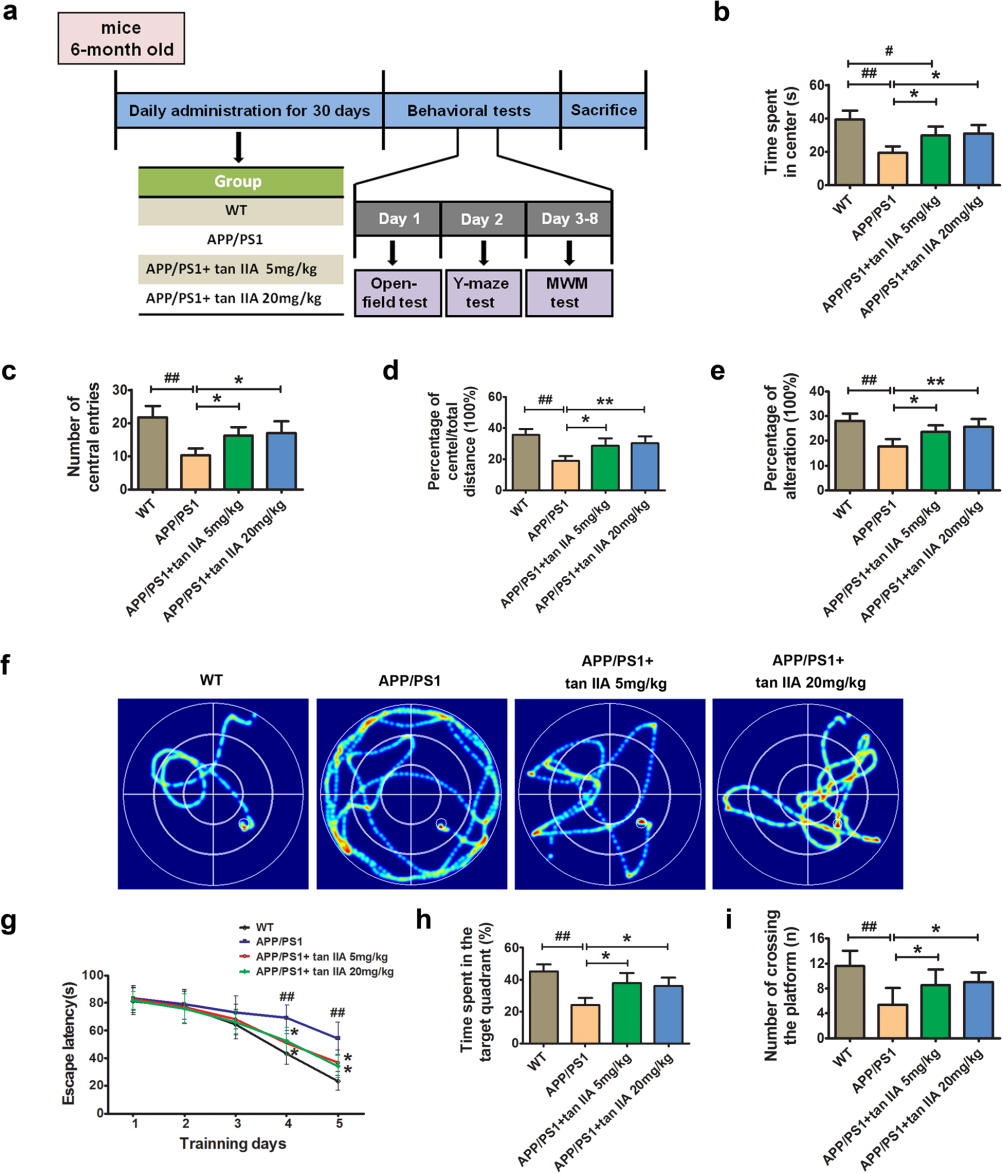
Tan IIA ameliorates learning and memory deficits in APP/PS1 mice. **a** Experimental design showing tan IIA administration in APP/PS1 mice and the behavioral tests. **b**–**d** Open-field test. Time spent in center **b**, number of central entries **c**, and relative distance in center **d**. **e** The percentage of spontaneous alternation in Y maze analysis. **f**–**i** Morris water maze test. Representative path tracings of each group during the navigation trials **f**, the escape latency **g**, the percentage of time spent in the target quadrant **h**, and the number of crossing through the platform **i**. *n* = 10. Data were presented as mean ± SD. ^*#*^*p* < 0.05 or ^*##*^*p* < 0.01 vs. WT group, and *p < 0.05 or **p < 0.01 vs. APP/PS1 group

Mice in tan IIA groups were intraperitoneally (i.p.) injected with tan IIA at a dose of 5 and 20 mg/kg once a day for 30 days, while mice in the control and APP/PS1 groups were administered with the same volume of NaCl for 30 days. In our preliminary experiments, 30 days administration of low-dose tan IIA (5, 10, and 20 mg/kg/day) was more effective than high-dose tan IIA (40 mg/kg/day) in rescuing behavioral deficits. To investigate the protective effects of different doses of tan IIA in Aβ accumulation, neuroinflammation, as well as synaptic and neuronal loss in APP/PS1 transgenic mice, tan IIA (5 and 20 mg/kg/day) was used in all experiments. The timeline for tan IIA administration and the behavioral tests are indicated in Fig. 1a.

### Behavioral analysis

#### Open-field test

To assess anxiety or motor performance deficits, the open-field test was performed. The apparatus consists of a large square chamber (40 cm × 40 cm × 40 cm). A camera was mounted centrally above the chamber and connected to a computerized video-tracking system (SMART, PanLab, Spain). Mice were removed from the cage and placed directly in the center of the chamber and allowed to freely explore for 5 min, without prior habituation. The time spent in the center of chamber (digitally designed by a 20 cm × 20 cm region), frequency of central entries, and the distance were recorded and analyzed by software. The chamber was cleaned with 70% ethanol to erase any scent cues and dried after every trial.

#### Y-maze test

Y-maze test is used to assess cognitive changes, short-term spatial working memory (by spontaneous alternation), and exploratory activity (by a total number of arm entries) of animals. The apparatus is a three-arm horizontal maze (30-cm long and 6-cm wide with 15-cm high walls) in which the arms are symmetrically disposed at 120° angles from each other. The maze wall and floor are constructed of dark gray plastic. In brief, mice were placed at the end of one arm and allowed to move freely through the maze for 8 min. All four paws must have been within the arm to be counted as an arm entry. A perfect alternation was defined as successive entries into the three arms on overlapping triplet sets. The number of total arm entries and the sequence of arm entries were recorded. The percentage of alternation (%) was calculated using the following formula: [successive triplet sets (consecutive entries into three different arms)/total number of arm entries − 2] × 100%. The maze was cleaned with 70% ethanol to remove residual odor after each test.

#### Morris water maze test

Spatial learning and reference memory ability were investigated using the Morris water maze (MWM) test. The maze consisted of a 120-cm diameter circular plastic pool filled with opaque water at 25 ± 2 °C during testing. The pool was divided into four quadrants equally named I, II, III, and IV, respectively. The MWM test lasted for 6 days as previously described [20, 24]. Briefly, mice were given orientation navigation test for 5 consecutive training days (4 trials a day). A hidden circular platform (diameter of 9 cm and a height of 30 cm) was placed under 1 cm of water in the middle of one quadrant (target quadrant) in all trials. For each daily trial, mice were randomly placed at one of the four equally spaced starting locations within the pool, and allowed to sit on the platform for 10 s after climbing onto it successfully. The maximum time allowed per trial was 90 s. If mice failed to locate the platform within 90 s, they were guided to the platform by the experimenter and kept there for 10 s. On the 6th day, for the probe trial, the platform was removed, and mice were allowed to swim freely in the pool for 90 s. The escape latency onto the platform, the time spent in the target quadrant, and the number of platform crossing times were recorded using a video camera and analyzed using the MWM software (Chengdu TME Technology Company, Chengdu, China).

### Brain tissue collection

Upon completion of behavior assays, mice were randomly selected and deeply anesthetized with 2% sodium pentobarbital and perfused transcardially with 0.9% NaCl followed by ice-cold 4% paraformaldehyde (PFA). The brain tissues were removed carefully and immediately postfixed in 4% PFA overnight at 4 °C, and then cryoprotected with 30% sucrose solution for 72 h. Afterward, the brains were embedded in optimum cutting temperature (OCT) compound and then cut into frozen serial coronal sections for morphological analysis. For biochemical assays, the remaining mice were perfused transcardially with 0.9% NaCl to clear blood from the body, and the cortex and hippocampus were dissected. Brain samples were immediately stored in a − 80 °C refrigerator until further assaying.

### Preparation of oligomeric Aβ_1–42_ and tan IIA

Preparation of oligomeric Aβ_1–42_ was based on a previously reported protocol [25, 29]. Briefly, 1 mg of lyophilized Aβ_1–42_ powder was dissolved in cold HFIP to a concentration of 1 mg/mL. The solution was evaporated, and the Aβ monomers were obtained. The peptide was resuspended in DMSO at a concentration of 2 mM and then diluted with DMEM to a final concentration of 5 μM and incubated at 4 °C for 24 h to prepare Aβ oligomers. Aβ_1-42_ oligomers were analyzed by dot blot and electron microscopy.

Tan IIA was dissolved in DMSO (no more than 0.1% in v/v) and was further diluted with DMEM.

### Cell culture and treatment

Murine microglia BV2 cells and human glioblastoma U87 cells were cultured in high glucose DMEM supplemented with 10% FBS, 100 units/mL penicillin, 100 μg/mL streptomycin, and 2 mM l-glutamine at 37 °C in a 5% CO_2_-humidified atmosphere. Once the confluence reached about 70–80%, cells were seeded at a density of 1.0 × 10^4^ cells/mL or 5.0 × 10^3^ cells/mL into 24-well and flasks and incubated overnight. The next day, BV2 and U87 cells were transferred to serum-free medium and then cultured in the presence or absence of tan IIA (1 and 10 μM) for 30 min, RAGE inhibitor (FPS-ZM1, 500 nM) for 48 h, or NF-κB inhibitor (Bay11-7082, 10 μM) for 30 min, followed by stimulation with oligomeric Aβ_1–42_ (5 μM) for another 24 h.

### Thioflavin S staining

Thioflavin S (Th-S) staining was performed as described previously [24, 30]. Briefly, the sections were washed with distilled water and stained in a 1% Th-S staining solution for 5 min. Then, the sections were differentiated in 70% ethanol for 1 min and mounted in 50% glycerin. The fluorescence image was detected by a fluorescence microscope. Th-S plaques were determined separately in the cortex and hippocampus. Quantitative analysis of Th-S staining area present in the sections was carried out under × 200 microscopic magnification and was counted on every five fields throughout the entire cortex and hippocampus by the Image-Pro Plus 6.0 software. The Aβ staining area (%) was calculated relative to the total area of the analyzed region (area% = plaque area/total area selected × 100%).

### Immunohistochemistry and immunofluorescence

For immunohistochemistry staining, the sections were rinsed in PBS and incubated in 3% hydrogen peroxide for 20 min and then blocked for nonspecific antigen binding using 1% BSA/0.3% Triton X-100 for 30 min at room temperature. Subsequently, the sections were incubated with specific antibodies against NeuN and MOAB2 at room temperature for 1 h and then overnight at 4 °C. On the next day, the sections were washed in PBS and incubated with the appropriate biotinylated secondary antibodies for 1 h at 37 °C. This was followed by incubation with HRP-conjugated streptavidin for 1 h at 37 °C. The staining was developed with 3, 3′-diaminobenzidine (DAB) for about 1–3 min. Finally, the sections were rinsed in TBS, dehydrated in graded ethanol, cleared in xylene, coverslipped with neutral balsam in a fume hood, and then observed under light microscopy.

For immunofluorescence staining, BV2 and U87 cells were fixed for 30 min using 4% PFA and permeabilized by 0.3% Triton X-100 for 15 min at room temperature. Next, the nonspecific antigen-binding sites were blocked by 1% BSA for 1 h at room temperature. Subsequently, the sections and cells were incubated with specific antibodies against GFAP, Iba-1, MOAB2, and NF-κB p65 at room temperature for 1 h and then overnight at 4 °C. After washing with PBS, the sections and cells were incubated with the appropriate secondary antibodies for 1 h at 37 °C. At last, the sections and cells were mounted and stained with DAPI, and the images were observed using a fluorescent microscope.

For quantification, six fields of the cortex and hippocampus were randomly selected from three different coronal sections of each mouse. GFAP-positive cells, Iba-1-positive cells, NeuN-positive cells, and the Aβ staining area (%) were counted and processed by observers who were blinded to the experiment design with the Image-Pro Plus 6.0 software.

### ELISA

To measure Aβ levels in APP/PS1 mouse brain, the cortical and hippocampal tissues were homogenized in cold tissue homogenization buffer containing protease inhibitor cocktail and centrifuged at 15,000 rpm for 1 h at 4 °C. After the supernatant (soluble fraction) was isolated, pellets were resuspended in guanidine buffer, rotated at room temperature for 2 h, and then, centrifuged at 15,000 rpm for 1 h at 4 °C. The supernatant was used as an insoluble fraction [30]. BV2 and U87 cells were cultured in 6-well culture plates respectively. After treatment, the culture media were collected and centrifuged at 8000 rpm for 30 min at 4 °C. The concentration of soluble/insoluble Aβ_1–40_ and Aβ_1–42_, TNF-α, IL-6, and IL-1β was measured using ELISA kits according to the manufacturer’s instructions. Absorbance was determined at 450 nm using a microplate absorbance reader.

### Real-time quantitative reverse-transcription PCR

Total mRNA was isolated and extracted from the mouse brain, BV2 cells, or U87 cells using RNAiso Plus according to the manufacturer’s protocol. Total mRNA (500 ng) was reverse-transcribed cDNA with the reverse transcription system. To quantify expression levels of genes, the cDNAs (2 μL) were amplified in real-time quantitative reverse-transcription PCR (qRT-PCR) using the following primers: mouse TNF-α (forward, 5′-GTC TAC TGA ACT TCG GGG TGA T-3′; reverse, 5′-ATG ATC TGA GTG TGA GGG TCT G-3′); mouse IL-6 (forward, 5′-ACA AAG CCA GAG TCC TTC AGA G-3′; reverse, 5′-CAT TGG AAA TTG GGG TAG GA-3′); mouse IL-1β (forward, 5′-GAA GAG CCC ATC CTC TGT GA-3′; reverse, 5′-ATG ATC TGA GTG TGA GGG TCT G -3′); mouse β-actin (forward, 5′-ACC ACA CCT TCT ACA ATG AG-3′; reverse, 5′-GGT TGG TGA AGT TGG TAG G-3′); human TNF-α (forward, 5′-CTG TGA AGG GAA TGG GTG TT-3′; reverse, 5′-CAG GGA AGA ATC TGG AAA GGT C-3′); human IL-6 (forward, 5′-GAG AGC ATT GGA AGT TGG GG-3′; reverse, 5′-CTT CCA GCC AGT TGC CTT CT-3′); human IL-1β, (forward, 5′-TGT GAC GTT CCC ATT AGA CAG -3′; reverse, 5′-GCT TGT GAG GTG CTG ATG TA-3′); human GAPDH, (forward, 5′-CTA GGC CAC AGA ATT GAA AGA TCT-3′; reverse, 5′-GTA GGT GGA AAT TCT AGC ATC ATC C′-3′). qRT-PCR was performed using SYBR® Premix Ex Taq™ II on a fluorescence thermocycler iQ5 (Bio-Rad). β-actin and GAPDH were used as endogenous controls. The relative levels of mRNA were analyzed using the 2^−ΔΔCt^ method.

### Western blotting

Briefly, the mouse brain, BV2 cells, or U87 cells were homogenized in ice-cold extraction reagent containing protease and phosphatase inhibitor cocktails. Next, the extract was centrifuged at 4 °C at 15,000 rpm for 30 min, and the supernatant was collected. Protein concentration was determined by the BCA protein assay reagents.

Equivalent amounts of protein (20 μg) for each sample were denatured by boiling at 95 °C for 7 min, and then were separated on 12–15% SDS-polyacrylamide gels. After electrophoresis, proteins were transferred to NC membranes. Membranes were blocked in 10% nonfat dry milk at room temperature for 1 h, then incubated at 4 °C overnight with primary antibodies against Syn, PSD-95, Iba-1, GFAP, RAGE, phospho-IκBα, total-IκBα, phospho-NF-κB p65, total-NF-κB p65, β-actin, and histone H3. After washing several times in TBST (tris-buffered saline containing 0.1% Tween 20), membranes were incubated with corresponding secondary antibodies for 2 h at room temperature. Blots were visualized using an enhanced ECL kit and analyzed using the Image J software.

### Statistical analysis

Results were expressed as mean ± standard deviation (SD) and analyzed with the SPSS 13.0 software. For water maze analysis of latency, two-way repeated measures analysis of variance (ANOVA) with Fisher’s LSD post hoc test was used to compare multiple groups. All other data were analyzed using one-way ANOVA with Tukey’s post hoc test. Statistical significance was assumed at *p* < 0.05.

## Results

### Tan IIA ameliorates learning and memory deficits in APP/PS1 mice

To investigate whether tan IIA treatment ameliorates learning and memory deficits in APP/PS1 mice, open-field test, Y-maze test, and MWM test were performed. The open-field test is mostly used to evaluate the exploration and anxiety-related behaviors in laboratory animals [31]. Compared with the WT group, APP/PS1 mice showed anxiety and less movement in exploring the central zone as indicated by less time spent in the center, fewer numbers of central entries, and shorter distance in the center. Interestingly, tan IIA treatment (5 and 20 mg/kg) significantly reversed this impairment behavior in APP/PS1 mice (Fig. 1b–d). After that, the Y-maze test, which evaluates spatial working memory, showed that the APP/PS1 group exhibited a markedly lower alternation behavior than the WT group. Following treatment with tan IIA treatment (5 and 20 mg/kg), this alternation behavior deficits were significantly ameliorated, demonstrating that tan IIA was effective in enhancing spatial working memory (Fig. 1e). Finally, the mice were subjected to MWM for consecutive 6 days to evaluate their spatial learning and memory. During the navigation testing, there were no obvious differences among the four groups on 1–3 days. On the 4th and 5th day, the APP/PS1 group had a higher escape latency than the WT group, whereas the escape latency in the tan IIA groups (5 and 20 mg/kg) was notably reduced, compared with the APP/PS1 group (Fig. 1f, g). On day 6, mice were subjected to a probe trial to evaluate their memory retention. As shown in Fig. 1 h and i, the lesser time spent in the target quadrant and decreased frequency of crossing the former platform location occurred in the APP/PS1 group when compared with the WT group. However, tan IIA treatment (5 and 20 mg/kg) showed better memory retention in the probe trial. Thus, these results suggested that tan IIA can mitigate the memory and cognitive impairment in AD development.

### Tan IIA prevents the neuronal and synaptic loss in APP/PS1 mice

**Fig. 2:**
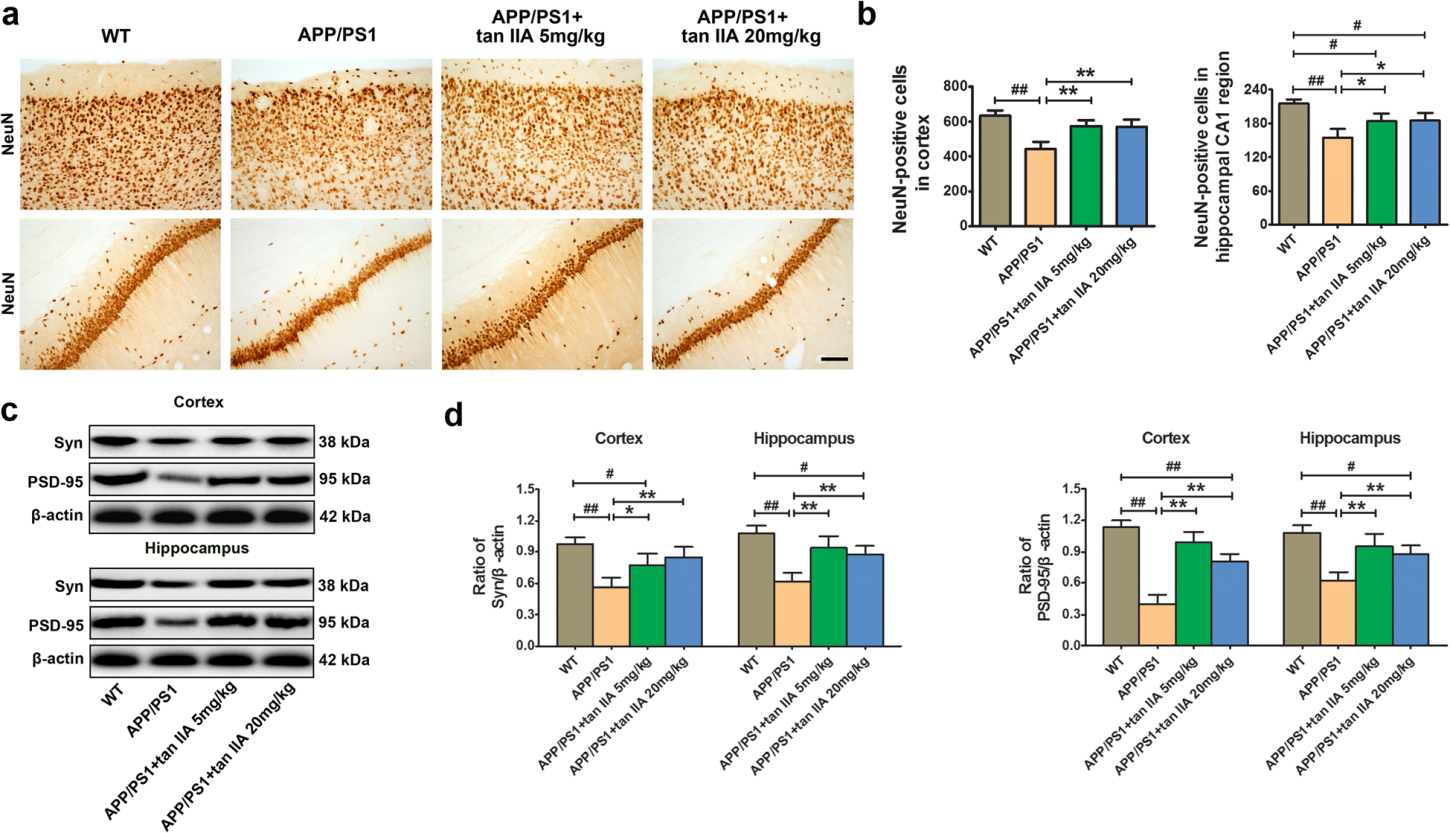
Tan IIA prevents the neuronal and synaptic loss in APP/PS1 mice. **a** Representative images of NeuN-positive cells in brain. Scale bar = 200 μm. **b** Quantitative analysis of NeuN-positive cells in the parietal cortex and hippocampal CA1 region. **c**, **d** Western blotting **c** and densitometry analysis **d** of Syn and PSD-95 in the parietal cortex and hippocampus. *n* = 5. Data were presented as mean ± SD. ^*#*^*p* < 0.05 or ^*##*^*p* < 0.01 vs. WT group, and *p < 0.05 or **p < 0.01 vs. APP/PS1 group

Since it is well-known that neuronal degeneration and loss is widely considered as the main contributors to cognitive impairment in AD [32, 33], to assess whether tan IIA can attenuate neuronal loss, we measured the change in the number of NeuN-positive neurons in the brain of APP/PS1 mice. Immunohistochemical staining showed that tan IIA treatment (5 and 20 mg/kg) prevented the loss of NeuN-positive neurons in the parietal cortex and hippocampal cornu ammonis 1 (CA1) region of APP/PS1 mice (Fig. 2a, b). The loss of synapses in the cortex and hippocampus correlates well with the cognitive decline [34]. To determine whether tan IIA improves synaptic impairment, western blotting was performed. The results showed that compared with the WT group, the levels of pre-synaptic protein Syn and post-synaptic protein PSD-95 in the brain of the APP/PS1 group were significantly decreased. However, tan IIA treatment (5 and 20 mg/kg) significantly reduced loss of Syn and PSD-95 in the parietal cortex and hippocampus (Fig. 2c, d). Collectively, these findings indicated that tan IIA delays or prevents neurodegenerative changes in APP/PS1 mice.

### Tan IIA reduces Aβ plaques and levels in APP/PS1 mice

**Fig. 3:**
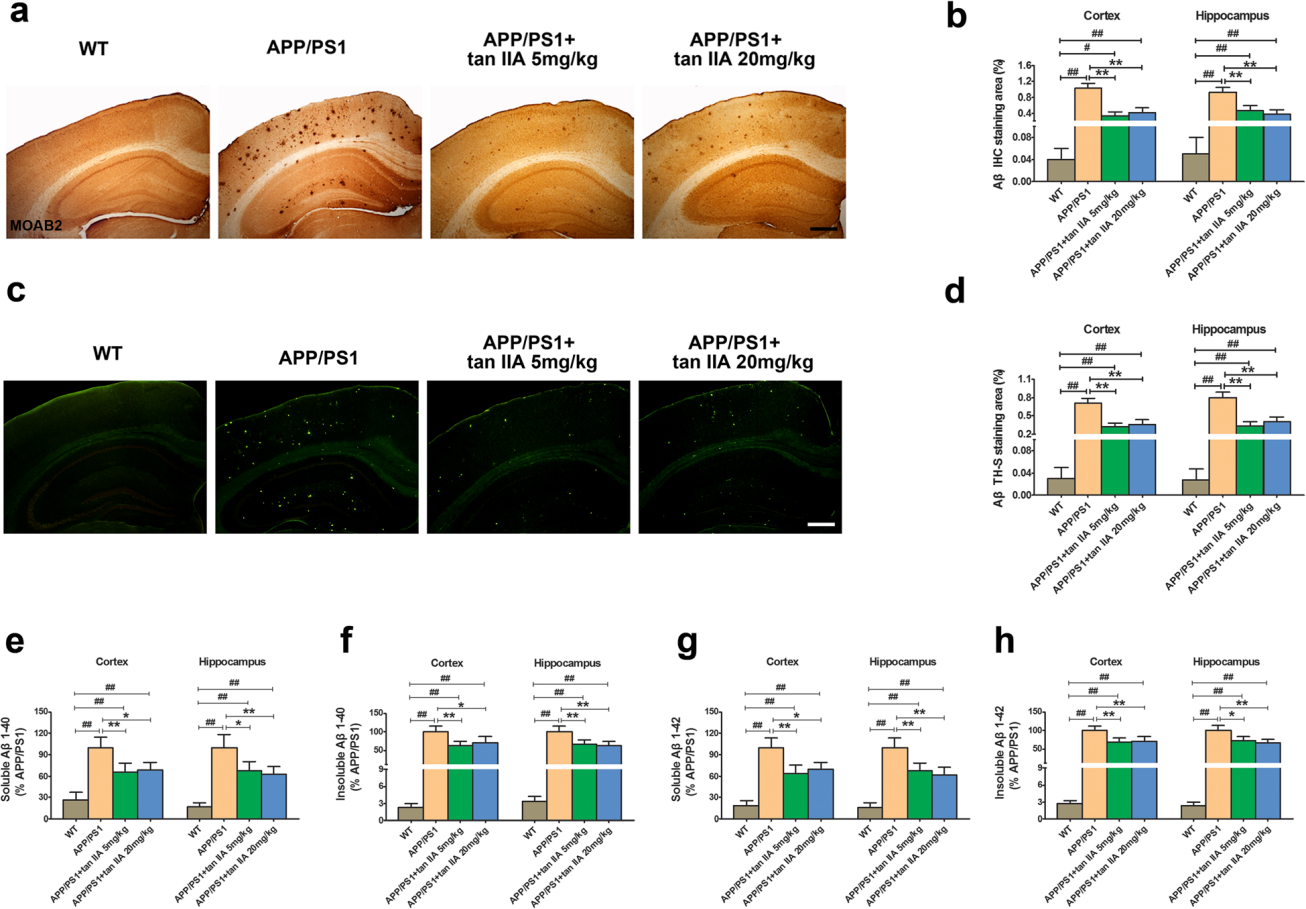
Tan IIA reduces Aβ plaques and levels in APP/PS1 mice. **a** Representative immunohistochemistry (IHC) staining of MOAB2 (Aβ plaques) in the brain. Scale bar = 200 μm. **b** Quantitative analysis of Aβ IHC staining area in the parietal cortex and hippocampus. **c** Representative Th-S-positive Aβ plaques in the brain. Scale bar = 200 μm. **d** Quantitative analysis of Aβ Th-S staining area in the parietal cortex and hippocampus. **e**–**h** The protein levels of soluble Aβ_1–40_ **e**, insoluble Aβ_1–40_ **f**, soluble Aβ_1–42_ **g**, and insoluble Aβ_1–42_ **h** in the parietal cortex and hippocampus. *n* = 5. Data were presented as mean ± SD. ^*#*^*p* < 0.05 or ^*##*^*p* < 0.01 vs. WT group, and *p < 0.05 or **p < 0.01 vs. APP/PS1 group

To determine whether tan IIA reduces Aβ load in the mouse brain, immunohistochemistry, Th-S staining, and ELISA were performed. As shown in Fig. 3 a and b, Aβ plaques were stained with a specific anti-Aβ antibody MOAB2, which were distributed throughout the cortex and hippocampus in the APP/PS1 group compared to the WT group. After tan IIA treatment (5 and 20 mg/kg), the percentage of the area occupied by immunostained plaques in the parietal cortex and hippocampus were markedly decreased. Consistent with the immunostaining results, Th-S staining also displayed a similar pattern of reduction in Aβ plaque area (Fig. 3c, d). Moreover, higher levels of soluble/insoluble Aβ_1–40_ in the parietal cortex and hippocampus were observed in the APP/PS1 group. Tan IIA treatment (5 and 20 mg/kg) significantly prevented the elevation of soluble/insoluble Aβ_1–40_ (Fig. 3e, f). Besides Aβ_1–40_, the soluble/insoluble Aβ_1–42_ in the parietal cortex and hippocampus were also dramatically reduced (Fig. 3g, h). Overall, these data demonstrated that tan IIA effectively reduces Aβ burden in APP/PS1 mice.

### Tan IIA suppresses microglial and astrocytic activation in APP/PS1 mice

**Fig. 4:**
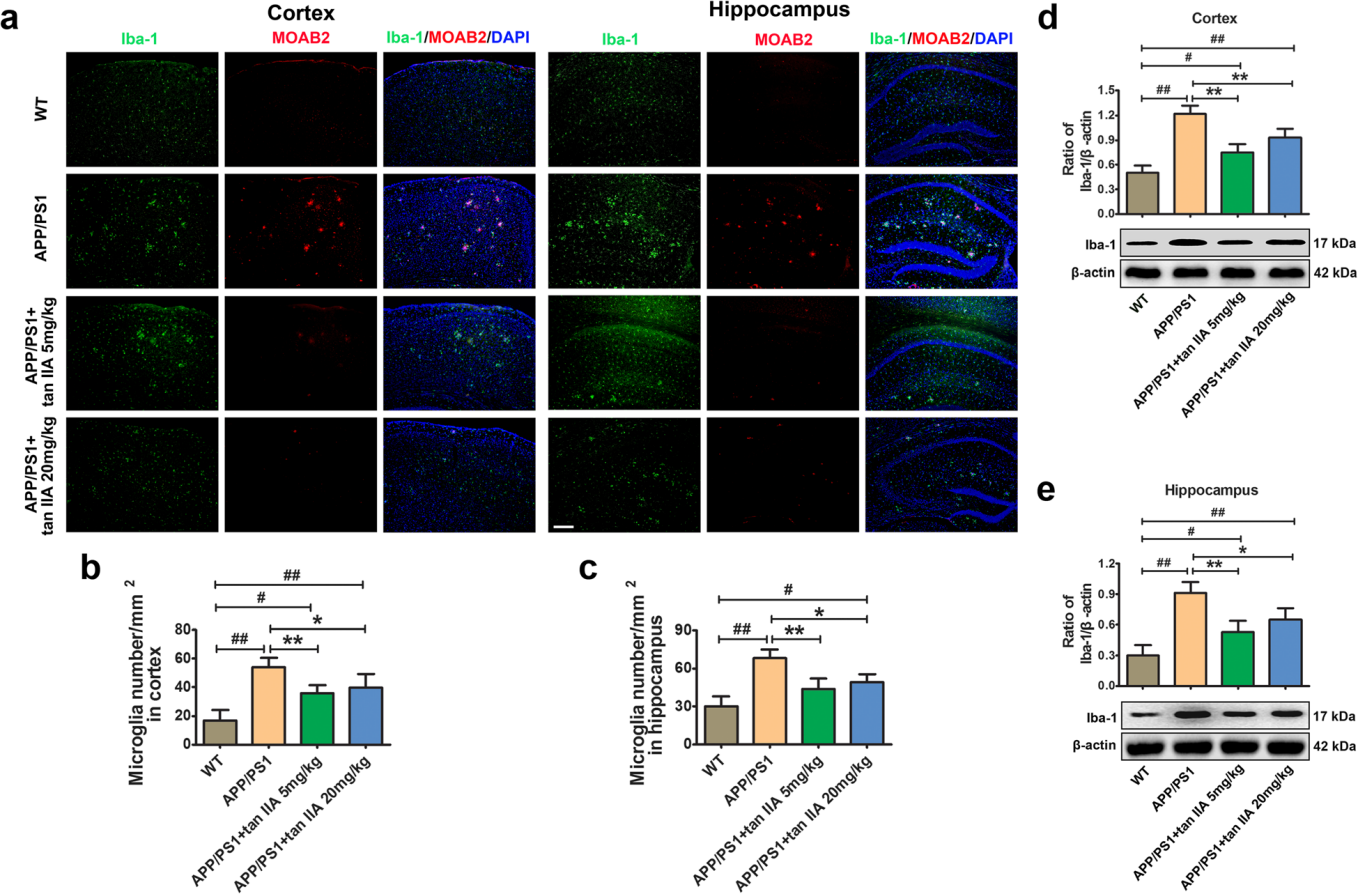
Tan IIA suppresses microglial activation in APP/PS1 mice. **a** Colocalization of fluorescent of Iba-1 (green), MOAB2-positive Aβ (red), and DAPI (blue) in the parietal cortex and hippocampus. Scale bar = 200 μm. **b** Quantitative analysis of microglia number in the parietal cortex. **c** Quantitative analysis of microglia number in the hippocampus. **d** Western blotting and densitometry analysis of Iba-1 in the parietal cortex. **e** Western blotting and densitometry analysis of Iba-1 in the hippocampus. *n* = 5. Data were presented as mean ± SD. ^*#*^*p* < 0.05 or ^*##*^*p* < 0.01 vs. WT group, and *p < 0.05 or **p < 0.01 vs. APP/PS1 group

**Fig. 5:**
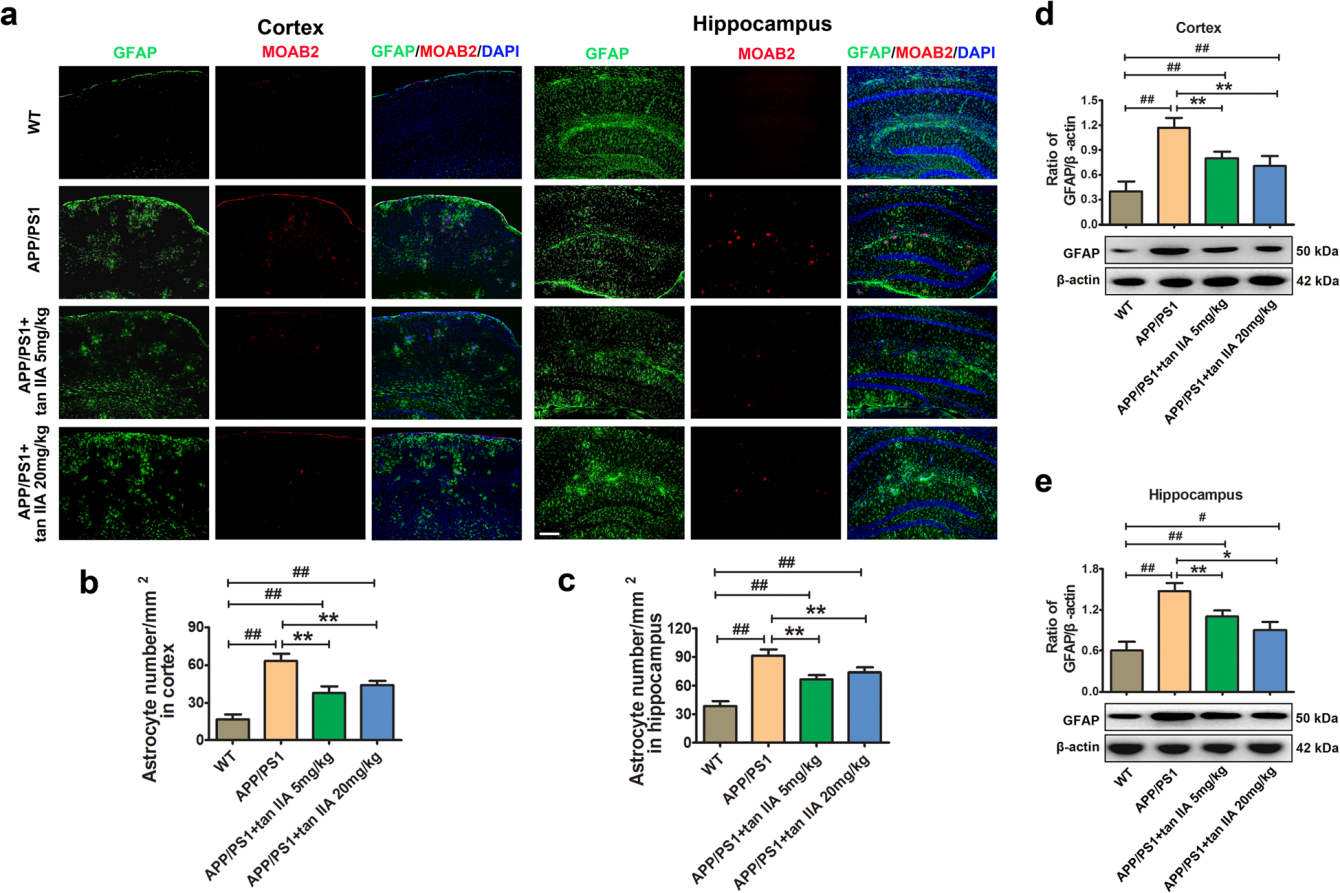
Tan IIA suppresses astrocytic activation in APP/PS1 mice. **a** Colocalization of fluorescent of GFAP (green), MOAB2-positive Aβ (red), and DAPI (blue) in the parietal cortex and hippocampus. Scale bar = 200 μm. **b** Quantitative analysis of astrocyte number in the parietal cortex. **c** Quantitative analysis of astrocyte number in the hippocampus. **d** Western blotting and densitometry analysis of GFAP in the parietal cortex. **e** Western blotting and densitometry analysis of GFAP in the hippocampus. *n* = 5. Data were presented as mean ± SD. ^*#*^*p* < 0.05 or ^*##*^*p* < 0.01 vs. WT group, and *p < 0.05 or **p < 0.01 vs. APP/PS1 group

An invariant feature of the AD brain is the presence of activated microglia and astrocytes surrounding Aβ plaques, which contributes to the neuroinflammatory responses and further exaggerating AD processes. To investigate the anti-inflammatory effects of tan IIA on APP/PS1 mice, immunofluorescence and western blotting were conducted. As shown in Fig. 4a-c, double-staining of Iba-1/Aβ (MOAB2) demonstrated a stronger presence of Iba-1-positive microglia in the area surrounding Aβ deposits in the APP/PS1 group, whereas tan IIA treatment (5 and 20 mg/kg) reduced the number of activated microglia in the parietal cortex and hippocampus. Meanwhile, western blotting revealed that Iba-1 protein levels in the brain of tan IIA groups (5 and 20 mg/kg) were markedly reduced compared to the APP/PS1 group (Fig. 4d, e). Further, double-staining of GFAP/Aβ (MOAB2) revealed that Aβ plaques were surrounded by reactive astrocytes in the brain of the APP/PS1 group compared with the WT group. As expected, tan IIA treatment (5 and 20 mg/kg) significantly decreased the number of GFAP-positive astrocytes, and the GFAP protein levels in the parietal cortex and hippocampus (Fig. 5). The above findings demonstrated that tan IIA effectively suppresses microglial and astrocytic activation, which subsequently attenuates microgliosis and astrogliosis in APP/PS1 mice.

### Tan IIA decreases pro-inflammatory cytokine production in APP/PS1 mice

**Fig. 6:**
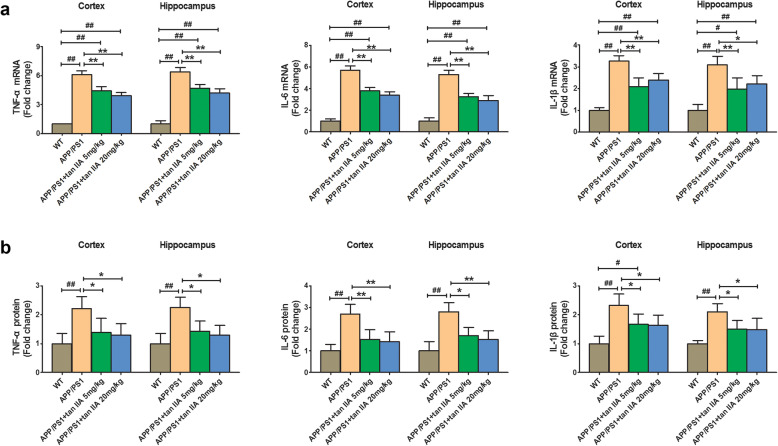
Tan IIA decreases pro-inflammatory cytokine production in APP/PS1 mice. **a** The mRNA expression of TNF-α, IL-6, and IL-1β in the parietal cortex and hippocampus were detected by qRT-PCR. **b** The protein expression of TNF-α, IL-6, and IL-1β in the parietal cortex and hippocampus were measured by ELISA. *n* = 5. Data were presented as mean ± SD. ^*#*^*p* < 0.05 or ^*##*^*p* < 0.01 vs. WT group, and *p < 0.05 or **p < 0.01 vs. APP/PS1 group

Activated microglia and astrocytes stimulated by Aβ play an important role in the release of pro-inflammatory chemokines and cytokines, which can lead to an increase in Aβ production, neuronal damage, and cognitive deficits [35]. Thus, to evaluate whether tan IIA can reduce the levels of pro-inflammatory cytokines in the brain of APP/PS1 mice, we measured the levels of TNF-α, IL-6, and IL-1β in the brain. As shown in Fig. 6a, the mRNA levels of TNF-α, IL-6, and IL-1β were markedly increased in the parietal cortex and hippocampus of the APP/PS1 group compared with the WT group. Interestingly, tan IIA treatment (5 and 20 mg/kg) significantly reduced the mRNA levels of TNF-α, IL-6, and IL-1β in both the parietal cortex and hippocampus. Also, to further confirm the above results, the protein levels of TNF-α, IL-6, and IL-1β were measured by ELISA. Results showed that the concentration of TNF-α, IL-6, and IL-1β in the brain was consistent with qRT-PCR results (Fig. 6b). Taken together, these data indicated that tan IIA could inhibit the secretion and expression of pro-inflammatory factors released by activated glial cells.

### Tan IIA inhibits RAGE/NF-κB signaling pathway activation in APP/PS1 mice

**Fig. 7:**
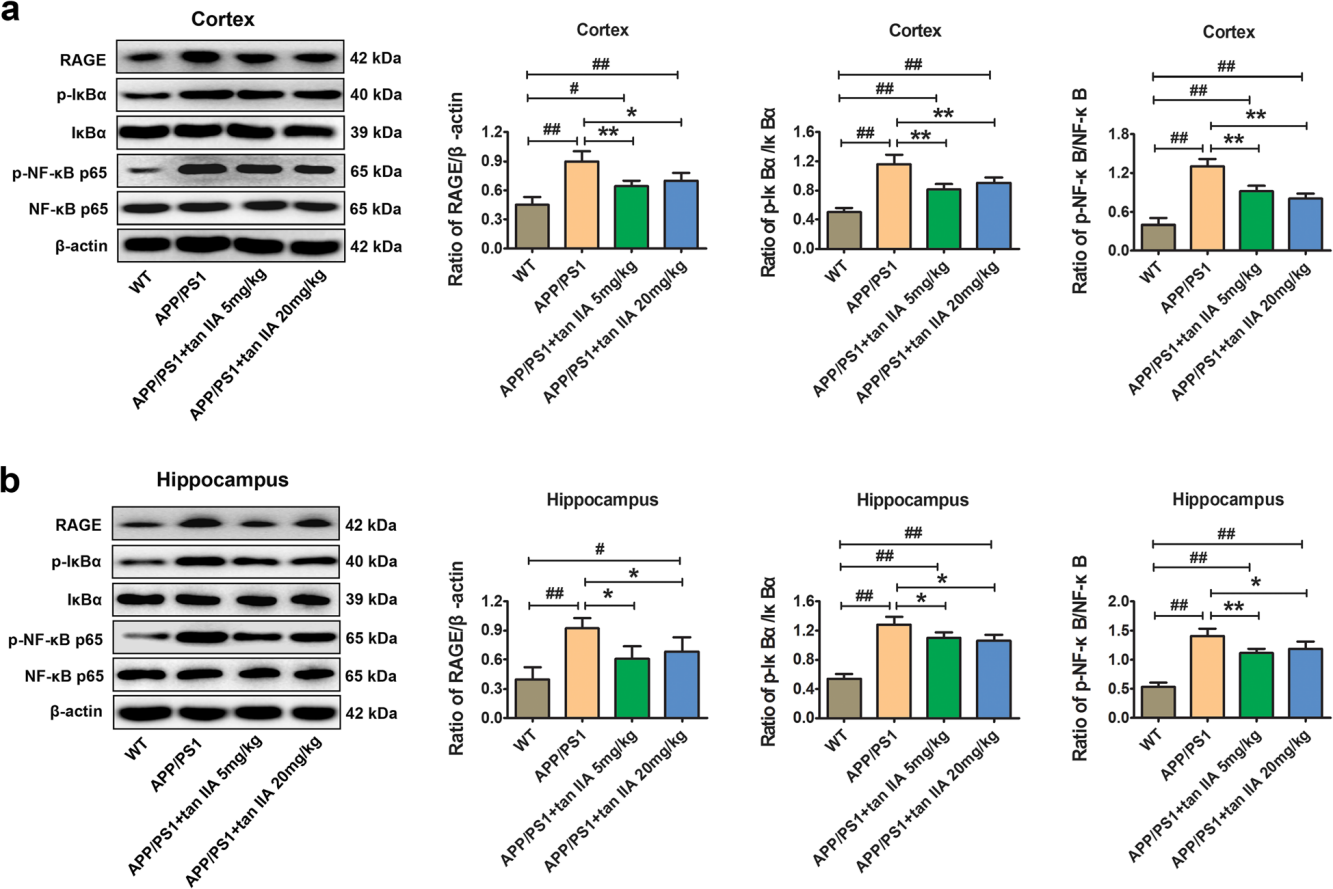
Tan IIA inhibits RAGE/NF-κB signaling pathway activation in APP/PS1 mice. **a** Western blotting and densitometry analysis of RAGE, phospho-IκBα, total-IκBα, phospho-NF-κB p65, and total-NF-κB p65 in the parietal cortex. **b** Western blotting and densitometry analysis of RAGE, phospho-IκBα, total-IκBα, phospho-NF-κB p65, and total-NF-κB p65 in the hippocampus. *n* = 5. Data were presented as mean ± SD. ^*#*^*p* < 0.05 or ^*##*^*p* < 0.01 vs. WT group, and *p < 0.05 or **p < 0.01 vs. APP/PS1 group

There is growing evidence that RAGE-mediated inflammatory pathways have been implicated in learning and memory deficits in AD [15, 36]. To examine whether tan IIA ameliorates neuroinflammatory responses by inhibiting the RAGE/NF-κB signaling pathway, the expression of RAGE-signaling molecules was determined by western blotting. The results showed that upon comparison with the WT group, the levels of RAGE, phospho-IκBα, and phospho-NF-κB p65 were increased in the parietal cortex and hippocampus of the APP/PS1 group, suggesting that the RAGE/NF-κB signaling pathway was activated. In contrast, tan IIA treatment (5 and 20 mg/kg) obviously reduced the expression of RAGE and the phosphorylation of IκBα and NF-κB p65 in the parietal cortex and hippocampus (Fig. 7). Therefore, it was indicated that tan IIA can downregulate the RAGE/NF-κB signaling pathway in APP/PS1 mice.

### Tan IIA suppresses RAGE/NF-κB signaling pathway activation in Aβ_1–42_-stimulated BV2 and U87 cells

**Fig. 8:**
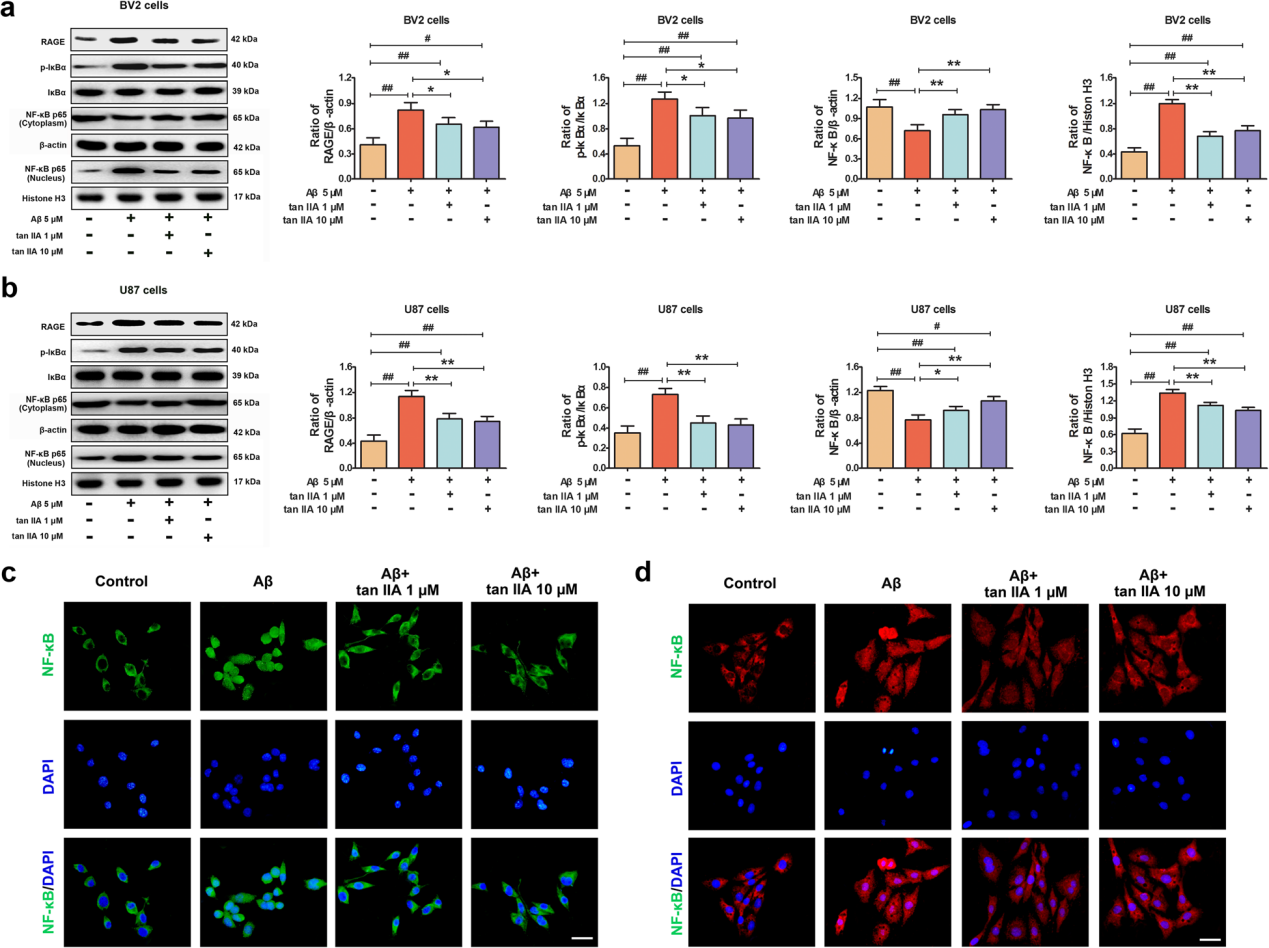
Tan IIA suppresses RAGE/NF-κB signaling pathway activation in Aβ_1–42_-stimulated BV2 and U87 cells. **a** Western blotting and densitometry analysis of RAGE, phospho-IκBα, total-IκBα, and total-NF-κB p65 (cytoplasm and nucleus) in BV2 cells. **b** Western blotting and densitometry analysis of RAGE, phospho-IκBα, total-IκBα, and total-NF-κB p65 (cytoplasm and nucleus) in U87 cells. **c** Immunofluorescence for NF-κB p65 (green) and DAPI (blue) in BV2 cells. Scale bar = 50 μm. **d** Immunofluorescence for NF-κB p65 (red) and DAPI (blue) in U87 cells. Scale bar = 50 μm. *n* = 5. Data were presented as mean ± SD. ^*#*^*p* < 0.05 or ^*##*^*p* < 0.01 vs. control, and *p < 0.05 or **p < 0.01 vs. Aβ_1–42_ treated cells

To further investigate whether tan IIA attenuates the Aβ_1-42_-induced neuroinflammatory responses by blocking the ligation of Aβ to RAGE and suppressing the RAGE-mediated NF-κB signaling pathway in glial cells. BV2 and U87 cells were pretreated with tan IIA (1 and 10 μM) for 30 min, followed by stimulation with oligomeric Aβ_1-42_ (5 μM) for 24 h. Dot blot and electron microscopy demonstrated that our preparation is a pure Aβ_1-42_ oligomer (Supplementary Fig. 1). As revealed by Fig. 8a, after treatment with Aβ1–42, the levels of RAGE and phospho-IκBα were significantly upregulated in BV2 cells. In addition, Aβ1–42 exposure decreased expression of NF-κB p65 subunit in the cytoplasm while increased its expression in the nucleus. As expected, pretreatment with tan IIA obviously decreased the levels of RAGE and phospho-IκBα, as well as suppressed the nuclear translocation of NF-κB p65 subunit. In support of this, immunofluorescence also showed that the levels of nucleocytoplasmic translocation of NF-κB p65 induced by Aβ_1–42_ were effectively suppressed by tan IIA treatment (Fig. 8c).

Next, we examined the effects of tan IIA on RAGE expression and the activation of the RAGE-signaling pathway in Aβ_1–42_-stimulated U87 cells. Similar to BV2 cell results, the RAGE/NF-κB signaling pathway was significantly activated by Aβ_1–42_, as increased the expression of RAGE and IκBα phosphorylation, and subsequently increased nuclear translocation of NF-κB p65 subunit. However, tan IIA effectively prevented this process, suggesting that tan IIA obviously inhibited NF-κB activation as well as the degradation of IκBα (Fig. 8b, d). Collectively, these data indicated that tan IIA achieves its anti-inflammation effects by blocking the RAGE/NF-κB signaling pathway in Aβ-stimulated BV2 and U87cells.

### Tan IIA, FPS-ZM1, and Bay11-7082 attenuate the production of pro-inflammatory cytokines in Aβ_1–42_-stimulated BV2 and U87 cells

**Fig. 9:**
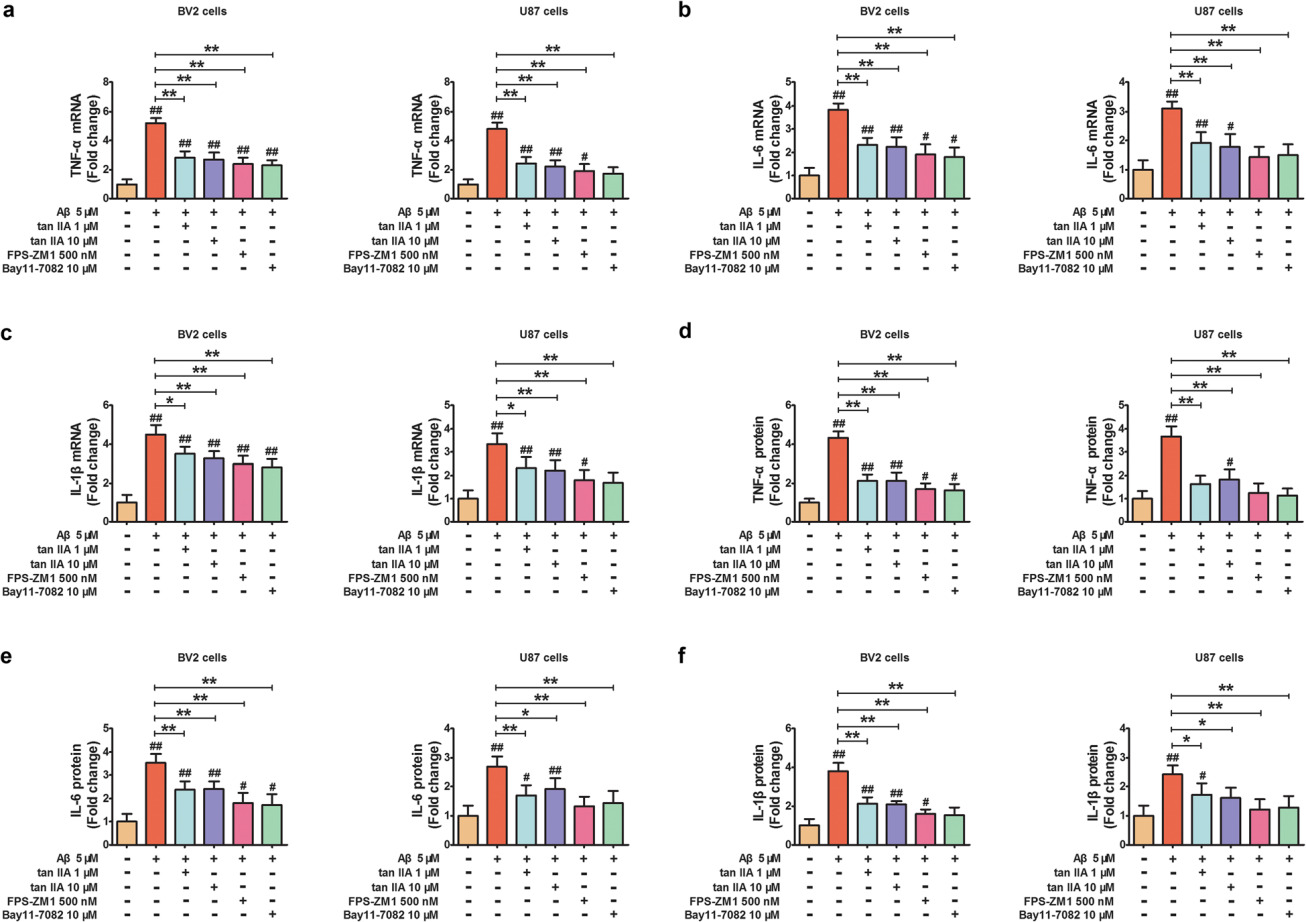
Tan IIA, FPS-ZM1, and Bay11-7082 attenuate the production of pro-inflammatory cytokines in Aβ_1–42_-stimulated BV2 and U87 cells. **a**–**c** The mRNA expression of TNF-α **a**, IL-6 **b**, and IL-1β **c** in BV2 and U87 cells were detected by qRT-PCR. **d**–**f** The protein expression of TNF-α **d**, IL-6 **e**, and IL-1β **f** in BV2 and U87 cells were measured by ELISA. *n* = 3. Data are presented as mean ± SD. ^*#*^*p* < 0.05 or ^*##*^*p* < 0.01 vs. control, and *p < 0.05 or **p < 0.01 vs. Aβ_1–42_ treated cells

Activated microglia and astrocytes are important mediators of Aβ-triggered neurotoxicity via the release of pro-inflammatory cytokines. To determine the potential regulatory effects of tan IIA on the production of pro-inflammatory cytokines in Aβ_1–42_-stimulated glial cells, BV2 and U87 cells were pretreated with tan IIA (1 and 10 μM) for 30 min, followed by stimulation with oligomeric Aβ (5 μM) for 24 h. Compared with the control, Aβ_1–42_ significantly increased the mRNA levels of TNF-α, IL-6, and IL-1β in BV2 and U87 cells. However, these increases were remarkably abrogated by tan IIA. Simultaneously, the protein levels of TNF-α, IL-6, and IL-1β in the culture medium showed a similar trend. To further investigate the molecular mechanism by which tan IIA affected the neuroinflammatory responses, BV2 and U87 cells were cultured in the presence of 500 nM FPS-ZM1 (RAGE inhibitor) for 48 h or 10 μM Bay11-7082 (NF-κB inhibitor) for 30 min, followed by stimulation with 5 μM Aβ_1–42_ for another 24 h. The results showed that the mRNA and protein levels of TNF-α were decreased by pretreatment with FPS-ZM1 and Bay11-7082. Meanwhile, a corresponding reduction in the expression of IL-6 and IL-1β was also observed (Fig. 9). Based on the above data, we concluded that tan IIA inhibits pro-inflammatory cytokine production by inhibiting the RAGE/NF-κB signaling pathway activation in Aβ-stimulated BV2 and U87cells.

## Discussion

**Fig. 10:**
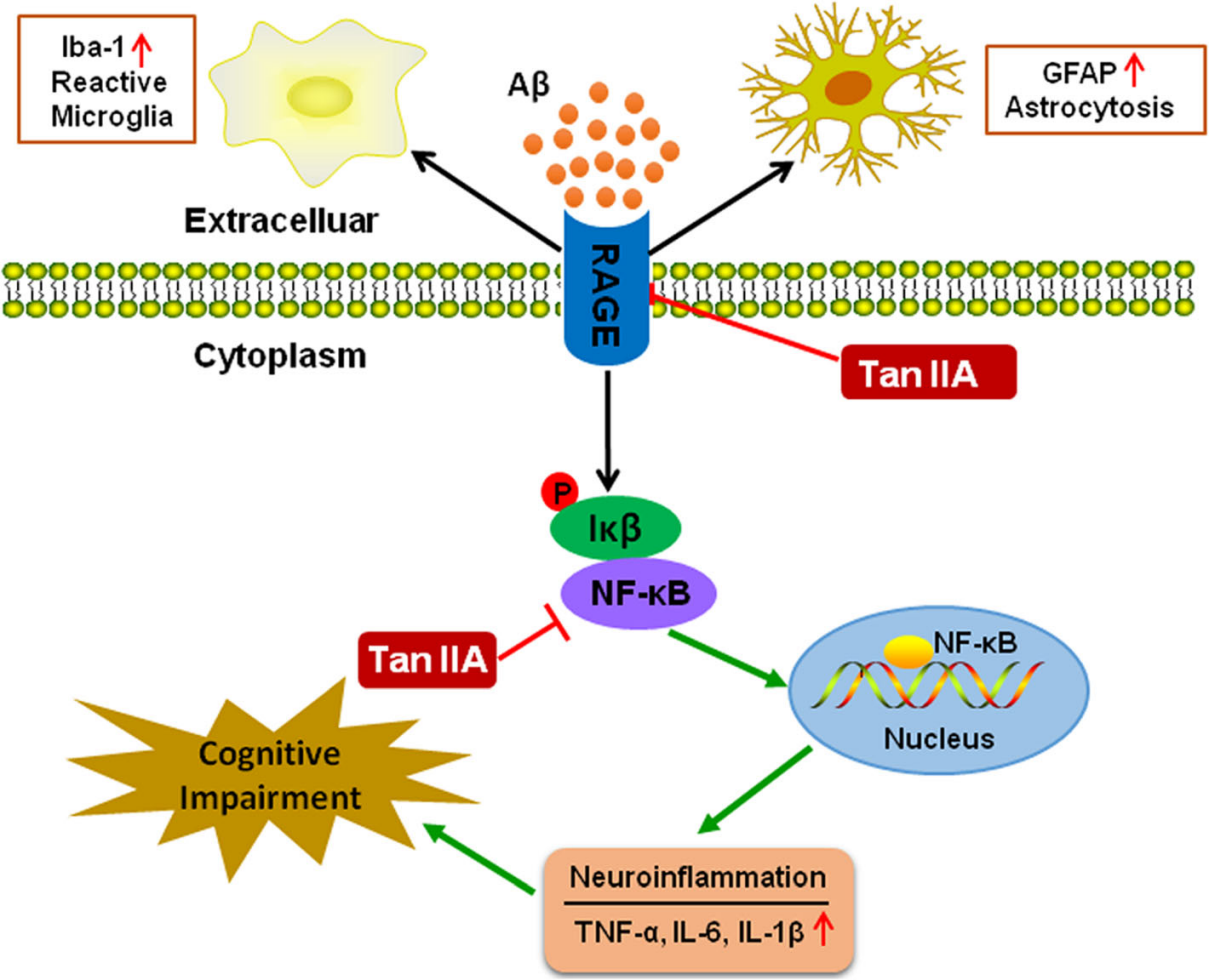
Diagram shows that tan IIA could rescue cognitive impairment, inhibit microglial and astrocytic activation, and decrease neuroinflammation. The mechanisms underlying the anti-neuroinflammation effects of tan IIA may involve the suppression pro-inflammatory cytokine production through the RAGE/NF-κB signaling pathway

Several published articles have demonstrated that tan IIA plays a protective role against inflammation and oxidative stress [21, 26–28]. However, its anti-inflammation effects and protective mechanisms in APP/PS1 mice are still largely unclear. In the present study, tan IIA treatment efficiently ameliorated cognitive deficits, neuronal and synaptic loss, Aβ accumulation, as well as neuroinflammation in APP/PS1 mice. The further mechanistic study revealed that tan IIA reduced the neuroinflammatory responses via the RAGE/NF-κB signaling pathway, as the number of activated microglia and astrocytes, the pro-inflammatory cytokines TNF-α, IL-6 and IL-1β, and the expression of RAGE-signaling molecules in the brain of APP/PS1 mice were significantly reduced (Fig. 10). Furthermore, the findings indicated that tan IIA attenuated Aβ_1–42_-induced release of pro-inflammatory cytokines via inhibiting the RAGE/NF-κB signaling pathway in BV2 and U87 cells.

Neuroinflammation, driven mainly by activated microglia and astrocytes, is an early event and a critical pathological feature in the pathogenesis of AD [2]. Increasing evidence indicated that neuroinflammatory responses were closely associated with the progression of several AD-related neuropathological alterations, including Aβ deposition, neuronal dysfunction, tau pathology, and memory impairment [37, 38]. Activated microglia and astrocytes produce a wide spectrum of pro-inflammatory cytokines and other mediators, which ultimately induce neuronal damage in the brain of AD. Therefore, the inhibition of glial cell activation is a key goal for the treatment of AD. In this study, we investigated whether tan IIA could suppress glial cell activation and inhibit neuroinflammatory responses in vivo. After treatment of tan IIA (5 and 20 mg/kg), the results of immunofluorescence staining revealed that the number of activated microglia and astrocytes was effectively reduced. Meanwhile, fewer Iba-1-positive microglia and GFAP-positive astrocytes were also observed around the less Aβ plaques in the brain of APP/PS1 mice. Furthermore, the protein levels of Iba-1 and GFAP were markedly decreased in the parietal cortex and hippocampus. To our knowledge, there are few reports regarding the effects of tan IIA on microgliosis and astrogliosis in AD. The current results were coincident with the previous study, which identified that tan IIA improves memory impairment could be through decreasing neuroinflammatory responses in the brain of AD mice [39].

Previous reports have indicated that RAGE expression is increased in microglia, neurons, and endothelial cells in the brain of patients with AD, leading to exacerbated AD pathogenesis in several mechanisms. First, RAGE enhances the production of Aβ and the aberrant hyperphosphorylation of tau. Second, it activates microglia and astrocytes into a reactive and inflammatory state. Third, it increases neurodegeneration and neuron loss and speed up the age-related cognitive impairment [9, 16]. Meanwhile, Aβ plaques and NFTs will further activate microglia and astrocytes and thus worsens the pathogenic progress of AD with a vicious cycle of Aβ plaques and NFTs, neuroinflammation and cellular stress, and neuronal dysfunction [15, 40, 41]. It is well-identified that RAGE is a transmembrane receptor that binds a broad repertoire of ligands. Binding of Aβ to RAGE activates a signaling cascade that leads to the activation of the transcription factor NF-κB, which augments the transcription of various pro-inflammatory cytokines (TNF-α, IL-6, and IL-1β) and enhances microglia and astrocyte activation [7, 42]. TNF-α was suggested to be a key mediator of AD in neuroinflammation and Aβ production [43]. IL-6 is mainly synthesized and secreted by astrocytes [44]. Previous studies demonstrated that mutations in the IL-6 gene may increase the risk for AD [45, 46]. IL-1β activates NF-κB and p38 signaling pathways to further worsen neuronal cell dysfunction [47]. Therefore, inhibiting the expression of RAGE and RAGE/NF-κB mediated neuroinflammatory responses may be an effective therapeutic strategy for AD. In the current study, the expression of RAGE and the phosphorylation of IκBα and NF-κB p65 in the parietal cortex and hippocampus were effectively decreased after treatment of tan IIA (5 and 20 mg/kg) in APP/PS1 mice. In addition, the pro-inflammatory cytokines TNF-α, IL-6, and IL-1β were also reduced. Furthermore, tan IIA (1 and 10 μM) suppressed the production of pro-inflammatory cytokines, the levels of RAGE and phospho-IκBα, and the nuclear translocation of NF-κB p65 subunit in Aβ_1–42_-stimulated BV2 and U87 cells, similar to the results of in vivo studies. Afterward, we attempted to determine the possible mechanisms underlying the inhibition of pro-inflammatory cytokine production in glial cells by tan IIA, RAGE inhibitor (FPS-ZM1), and NF-κB inhibitor (Bay11-7082) used. We found that the levels of TNF-α, IL-6, and IL-1β were markedly decreased by pretreatment with FPS-ZM1 and Bay11-7082 in BV2 and U87 cells. The above results suggested that tan IIA may attenuate the expression of pro-inflammatory cytokines, at least partially via inhibition of the RAGE/NF-κB signaling pathway.

Evidence has shown that neuroinflammation is related to the accumulation of Aβ in the brain of AD [48, 49]. Abnormal Aβ deposition is widely considered to play a pivotal role in AD. Therefore, suppressing neuroinflammation could be a promising treatment to prevent or reduce Aβ aggregation. In this study, APP/PS1 mice showed less Aβ plaques in the parietal cortex and hippocampus after treatment of tan IIA (5 and 20 mg/kg). Additionally, the protein levels of both soluble/insoluble Aβ_1–40_ and Aβ_1–42_ were lower in the parietal cortex and hippocampus. It has been reported that tan IIA could alleviate pathological symptoms of AD via decreasing the accumulations of Aβ [24, 50, 51]. Thus, taken together with the above findings, we concluded that tan IIA-inhibited neuroinflammation may be another mechanism by which tan IIA reduces Aβ burden in APP/PS1 mice. However, further studies are needed to explore the mechanism and potential effects of tan IIA on Aβ aggregation.

Previous studies reported that neuronal and synaptic loss in the cortex and hippocampus correlated best with cognitive dysfunction in AD, indicating that it is important in the pathogenesis [32, 33, 52]. The synapse-associated proteins, especially pre-synaptic Syn and post-synaptic PSD-95, play an important role in synaptic plasticity and memory formation [53–55]. It has been shown that pro-inflammatory cytokines are responsible for neuronal and synaptic loss, whereas anti-neuroinflammatory therapies may effectively attenuate synaptic damage and improve cognitive deficits [56]. The immunohistochemical staining showed that tan IIA treatment (5 and 20 mg/kg) remarkably prevented the neuronal loss in the parietal cortex and hippocampal CA1 region in APP/PS1 mice. In addition, the expression of Syn and PSD-95 in the parietal cortex and hippocampus was markedly increased. We then assessed the neuroprotective effects of tan IIA on spatial learning and memory using established behavioral tests. In the open-field test, APP/PS1 mice showed less anxiety and better exploratory after treatment of tan IIA (5 and 20 mg/kg). Furthermore, spatial working memory deficits were evaluated by the Y-maze test; a markedly increased percentage of spontaneous alteration was observed in tan IIA (5 and 20 mg/kg) groups. At last, in the hidden platform test, the escape latency was obviously decreased after tan IIA treatment (5 and 20 mg/kg). For the probe test, tan IIA groups showed a significant increase in the time spent in the target quadrant and the number of target crossing, which is consistent with our previous reports [20, 24]. Overall, these results presented here suggested that tan IIA may contribute to the prevention of neuronal and synaptic loss in the parietal cortex and hippocampus, as well as the improvement of cognitive impairment. In the future, the impact of tan IIA on memory impairments and AD-driven inflammation would be worth investigating in APP/PS1/tau mouse models like 3xTg or 5xFAD.

Although tan IIA had a positive effect at the doses used here, we and other groups found that tan IIA increased the cytotoxicity of cortical neurons and SH-SY5Y cells at higher doses [22, 25, 57]. Furthermore, tan IIA exhibited severe cardiotoxicity, development malformation, and growth inhibition at high concentrations in a zebrafish embryo model [58]. These studies demonstrate that tan IIA has cytotoxic effects at higher doses and point to the potential risk of clinical application of increased doses. In the current study, results showed that tan IIA treatment (5 and 20 mg/kg) appear to exert the same degree of benefit, suggesting that tan IIA plays a similar neuroprotective role at these doses. Whether lower doses are also beneficial remains to be determined.

## Conclusions

In summary, the present study demonstrated that tan IIA ameliorated the neuropathologies including synaptic and neuronal loss, gliosis, neuroinflammatory responses, and Aβ deposition, accompanied by an improved spatial cognitive function. Furthermore, the in vitro evidence showed that tan IIA reduced the pro-inflammatory cytokines production by suppressing the RAGE/NF-κB signaling pathway. Thus, this study indicated that tan IIA may provide a novel therapeutic approach to prevent cognitive decline and neuroinflammation in AD. The promising results obtained in this work need to be complemented with human studies to further investigate the potential application of tan IIA in preventing AD progression.

## Supplementary information

**Additional file 1: **Figure S1. Demonstration of Aβ_1-42_ oligomers by dot blot and electron microscopy **a** Dot blot analysis of the composition of Aβ_1-42_. 1 uL of Aβ_1-42_ was applied to a nitrocellulose membrane and probed with rabbit anti-oligomer antibody or with anti-amyloid fibril antibody. **b** Electron microscopy analysis of the structure of the Aβ_1-42_ aggregates. 20 uL of Aβ_1-42_ oligomer preparation was dropped onto a 300-mesh carbon nickelgrid, and after 5 min the solution was removed. Sample was stained for 2 min with phosphotungstic acid. The diameter of preparation is consistent with that of the oligomer diameter distribution.

## Data Availability

Not applicable.

## Abbreviations

AD: Alzheimer’s disease
Aβ: Amyloid beta
AGEs: Advanced glycation end products
ANOVA: Analysis of variance
BSA: Bovine serum albumin
DMEM: Dulbecco’s modified Eagle’s medium
DMSO: Dimethylsulfoxide
ECL: Enhanced chemiluminescence
ELISA: Enzyme-linked immunosorbent assay
GFAP: Glial fibrillary acidic protein
HFIP: Hexafluoro-2-propanol
HRP: Horseradish peroxidase
Iba-1: Ionized calcium-binding adapter molecule 1
IL-1β: Interleukin-1β
IL-6: Interleukin-6
MWM: Morris water maze
NC: Nitrocellulose filter
NFTs: Neurofibrillary tangles
NF-κB: Nuclear factor-κB
Tan IIA: Tanshinone IIA
Th-S: Thioflavin S
TNF-α: Tumor necrosis factor-α
OCT: Optimum cutting temperature
PFA: Paraformaldehyde
qRT-PCR: Real-time quantitative reverse-transcription polymerase chain reaction
RAGE: Receptor for advanced glycation end products
Syn: Synaptophysin
WT: Wild-type
