# Supplementary material for: Tanshinone IIA attenuates neuroinflammation via inhibiting RAGE/NF-κB signaling pathway in vivo and in vitro
Source: J Neuroinflammation. 2020 Oct 14;17:302. doi: 10.1186/s12974-020-01981-4 (PMC7559789; doi:10.1186/s12974-020-01981-4)
Supplement: Supplementary file 1 — Additional file 1: Figure S1. Demonstration of Aβ1-42 oligomers by dot blot and electron microscopy a Dot blot analysis of the composition of Aβ1-42. 1 uL of Aβ1-42 was applied to a nitrocellulose membrane and probed with rabbit anti-oligomer antibody or with anti-amyloid fibril antibody. b Electron microscopy analysis of the structure of the Aβ1-42 aggregates. 20 uL of Aβ1-42 oligomer preparation was dropped onto a 300-mesh carbon nickelgrid, and after 5 min the solution was removed. Sample was stained for 2 min with phosphotungstic acid. The diameter of preparation is consistent with that of the oligomer diameter distribution. [file 12974_2020_1981_MOESM1_ESM.zip › Supplementary.docx]

Additional file 1

Figure S1**.** Demonstration of Aβ_1-42_ oligomers by dot blot and electron microscopy **a** Dot blot analysis of the composition of Aβ_1-42_**.** 1 uL of Aβ_1-42_ was applied to a nitrocellulose membrane and probed with rabbit anti-oligomer antibody or with anti-amyloid fibril antibody. **b** Electron microscopy analysis of the structure of the Aβ_1-42_ aggregates. 20 uL of Aβ_1-42_ oligomer preparation was dropped onto a 300-mesh carbon nickelgrid, and after 5 min the solution was removed. Sample was stained for 2 min with phosphotungstic acid. The diameter of preparation is consistent with that of the oligomer diameter distribution.
